# *In Vitro vs In Silico* Detected SNPs for the Development of a Genotyping Array: What Can We Learn from a Non-Model Species?

**DOI:** 10.1371/journal.pone.0011034

**Published:** 2010-06-09

**Authors:** Camille Lepoittevin, Jean-Marc Frigerio, Pauline Garnier-Géré, Franck Salin, María-Teresa Cervera, Barbara Vornam, Luc Harvengt, Christophe Plomion

**Affiliations:** 1 INRA, UMR1202 BIOGECO, Cestas, France; 2 Université de Bordeaux, UMR1202 BIOGECO, Talence, France; 3 FCBA, Laboratoire de Biotechnologies, Nangis, France; 4 INIA, Departamento de Ecología y Genética Forestal, Madrid, Spain; 5 University of Goettingen, Goettingen, Germany; University of Umeå, Sweden

## Abstract

**Background:**

There is considerable interest in the high-throughput discovery and genotyping of single nucleotide polymorphisms (SNPs) to accelerate genetic mapping and enable association studies. This study provides an assessment of EST-derived and resequencing-derived SNP quality in maritime pine (*Pinus pinaster* Ait.), a conifer characterized by a huge genome size (∼23.8 Gb/C).

**Methodology/Principal Findings:**

A 384-SNPs GoldenGate genotyping array was built from i/ 184 SNPs originally detected in a set of 40 re-sequenced candidate genes (*in vitro* SNPs), chosen on the basis of functionality scores, presence of neighboring polymorphisms, minor allele frequencies and linkage disequilibrium and ii/ 200 SNPs screened from ESTs (*in silico* SNPs) selected based on the number of ESTs used for SNP detection, the SNP minor allele frequency and the quality of SNP flanking sequences. The global success rate of the assay was 66.9%, and a conversion rate (considering only polymorphic SNPs) of 51% was achieved. *In vitro* SNPs showed significantly higher genotyping-success and conversion rates than *in silico* SNPs (+11.5% and +18.5%, respectively). The reproducibility was 100%, and the genotyping error rate very low (0.54%, dropping down to 0.06% when removing four SNPs showing elevated error rates).

**Conclusions/Significance:**

This study demonstrates that ESTs provide a resource for SNP identification in non-model species, which do not require any additional bench work and little bio-informatics analysis. However, the time and cost benefits of *in silico* SNPs are counterbalanced by a lower conversion rate than *in vitro* SNPs. This drawback is acceptable for population-based experiments, but could be dramatic in experiments involving samples from narrow genetic backgrounds. In addition, we showed that both the visual inspection of genotyping clusters and the estimation of a *per* SNP error rate should help identify markers that are not suitable to the GoldenGate technology in species characterized by a large and complex genome.

## Introduction

In the last few years, the development of high-throughput methods for the detection and genotyping of single nucleotide polymorphisms (SNPs) has led to a revolution in their use as molecular markers [1]. Their abundance in animal and plant genomes, the reduction in cost and the increased throughput of SNP assays have made these markers attractive for high-resolution genetic mapping, fine mapping of QTLs, linkage-disequilibrium based association mapping, genetic diversity analyses, genotype identification, marker-assisted selection and characterization of genetic resources [2], [3], [4], [5], [6], [7].

In non-model species, large scale SNP genotyping involves two main steps: first the discovery of polymorphisms, and second the genotyping of a set of specimens. SNP identification can proceed either from *in vitro* or *in silico* approaches. *In vitro* methods, such as the re-sequencing of targeted amplicons, are generally more appropriate when sequence data is limited or when one is interested in polymorphisms in specific genotypes or candidate genes. This approach is generally costly and time consuming, but has been proven successful to detect SNPs in many organisms (reviewed by [8]). In contrast, *in silico* discovery is the most obvious method for *de novo* SNP identification. Although this approach mainly provides markers located in transcribed regions (mostly coding and 3′UTR), it offers a low cost source of abundant SNPs and has been validated by large scale genotyping for a number of plant species including *Arabidopsis* [9], maize [10], grapevine [11], melon [12], tomato [13], spruce [14] or pine [15]. However, the usefulness of EST resources for detecting *in silico* SNPs varies depending on the assembly depth, the range of tissues considered, the diversity of the target species, but also on how well this diversity is represented within the database [2], [16], [17]. The number of *in silico* SNPs available will thus differ considerably between species, although a global trend towards more SNPs for more ESTs from different tissues is expected for species with similar diversity. For example, about 9,000 high quality SNPs were detected in a first catfish assembly comprising 54,960 ESTs [18]; this number extended to 48,000 when using a second assembly of nearly 500,000 ESTs [19]. EST resources can also be very useful for closely related species when the assembly is performed with all ESTs together, since detection of interspecific *in silico* SNPs is then possible, as shown by Wang *et al.* [19] for blue and channel catfish species.

There is no one ideal method for SNP genotyping and the selection of an appropriate technique largely depends on many factors including cost, accuracy, multiplexing capacity and throughput, equipment and difficulty of assay development [20], [21]. A range of high-throughput methods are currently developed for model species such as humans, but their use in non-model species with large genome size, high level of ploidy or redundancy is often a challenge [22]. Recently, Pavy *et al.* [23] and Eckert *et al.* [24] achieved the multiplexed genotyping of hundreds of SNPs in conifers, a group of plants that is characterized by a large genome size [25]. They used the Illumina bead array platform combined with GoldenGate assay [26], [27]. This genotyping platform was also successfully used for genomes containing a high number of paralogous genes such as barley [28], soybean [29] or tetraploid and hexaploid wheat [30].

Maritime pine (*Pinus pinaster Ait.*) genome is extremely large (up to 23.8 Gb/C, which is 150 times larger than that of *Arabidopsis thaliana*) [25]. Despite the economical and ecological importance of this species in south-western Europe, where it covers over 4M ha, it will be many years before its full genome sequence is available. However, about 30,000 *P. pinaster* expressed sequence tags (ESTs) were produced in the past decade, followed by the re-sequencing of more than 40 wood-quality and drought-stress related candidate genes [31], [32]. We report here the valorization of these resources to the first highly multiplexed SNP genotyping array in *P. pinaster*. Our objectives were three-fold: i/ validate a number of SNPs for future linkage mapping and candidate-gene-based association studies, and ii/ compare the conversion rate of SNPs derived from *in vitro versus in silico* datasets, as to our knowledge no other study in conifers has attempted to genotype a large number of *in silico* SNPs without preliminary re-sequencing, and iii/ estimate the genotyping error rate of the GoldenGate technology for a conifer genome, which has not been reported so far. The SNPs validated in this study have been made available through the NCBI database (http://www.ncbi.nlm.nih.gov/SNP, see Table S1 for accession numbers).

## Materials and Methods

### Plant material

Plant material consisted of 456 individuals, including: 212 unrelated trees resulting from mass selection in the natural forest of south-western France (first-generation breeding population, referred as the “G0” Aquitaine population), 210 offspring resulting from open-pollinated or controlled crosses among the G0 trees (second-generation breeding population, referred as the “G1” Aquitaine population), 29 trees randomly sampled in the same geographical area as the G0 trees, and 5 trees involved in two- and three-generation outbreed pedigrees, used for linkage and QTL mapping. DNA was extracted from needles using Invisorb® Spin Plant Mini Kit (Invitek, Berlin, Germany), and quantified with a Nanodrop ND-1000 spectrophotometer (NanoDrop Technologies, LLC, Wilmington, DEL, USA).

### SNP discovery

For SNP discovery, two sets of sequences were considered. The first dataset comprised maritime pine sequences for 41 different genes involved in plant cell wall formation (candidate genes for wood quality) or drought stress resistance (Table S2). For each fragment, an average of 50 megagametophytes (haploid tissue surrounding the embryo) from different populations were sequenced. The chromatograms were visually checked (nucleotides with phred scores below 20 were considered as missing data) and the SNPs were considered as true. Indeed, the use of megagametophytes lowered the risk of confusing polymorphism at a unique locus with differences between paralogous loci, as amplification of two or even more members of a gene family would have been easily detected by the visualization of double peaks in the chromatograms. This first set of SNPs will be referred to as *in vitro* SNPs. The second sequence dataset consisted in a collection of 26,476 maritime pine ESTs assembled in 3,995 non-singleton contigs and 7003 singletons (unigene available online at http://cbi.labri.fr/outils/SAM2/COMPLETE/ under the project name “Pinus pinaster 14_02_2007”). These ESTs were derived from six different libraries constructed using different tissues, and a number of segregating haploid genomes from 3 up to 300 from different provenances (Table 1). We used the Polybayes software [33] to detect SNPs with a high probability with the parameters described for maritime pine in Le Dantec *et al.* [15]. This second set of SNPs will be referred to as *in silico* SNPs.

**Table 1:** cDNA library information.

Library	Tissue	Nb of haploid genomes	Maritime pine provenance	Nb of ESTs
GEMINI	Xylem	4	Corsica	8,129
Normal aerial parts (AN)	Needles	300	Aquitaine	240
Stressed aerial parts (AS)	Needles	300	Aquitaine	475
Normal roots (RN)	Roots	300	Aquitaine	4,592
Stressed roots (RS)	Roots	300	Aquitaine	4,274
Buds (LG0ACA)	Buds	Unknown	Spain	8,766
TOTAL				26,476

### SNP selection for array construction

We developed a Perl script, *snp2illumina*, for automatically extracting SNPs from multifasta sequence files and output them as a SequenceList file compatible with the Illumina Assay Design Tool software (available online at http://www.illumina.com). This file contains the SNP names and surrounding sequences with polymorphic loci indicated by IUPAC codes for degenerated bases. The Perl script *snp2illumina* can work in batch mode and is available upon request from the corresponding author.

The functionality score provided by Assay Design Tool software is similar to a predicted probability of genotyping success, taking into account the sequence conformation around the SNP, the lack of repetitive elements in the surrounding sequence, and in the case of model species the sequence redundancy against the available sequence database [34]. In the case of maritime pine, no sequence database was available to test for sequence redundancy. All the SNPs presenting a functionality score below 0.4, which is considered as a lower limit for genotyping success by the manufacturer, were discarded.

Two contrasted strategies “depth vs. breath of SNP coverage” were adopted to select informative SNPs. In respect to *in vitro* SNPs, our objective was to include as many polymorphisms as possible for each gene fragment so depth of coverage was preferred. For *in silico* SNPs, our goal was to include a low number of markers per unigene in a large number of unigenes, thus giving more emphasis to breath of coverage. The main technical constraint for selecting *in vitro* SNPs was that the selected polymorphisms should not be less than 60 nucleotides away from each other. When several SNPs stood within this limit it was decided to filter out lowest frequency variants and polymorphisms showing high level of linkage disequilibrium with other selected SNPs of the same fragment. Rare variants (minor allele frequency <5%) were also discarded. To select *in silico* SNPs we used the log-file of the *snp2illumina* script that records for each SNP the number of ESTs considered for the detection, the minor allele frequency (MAF) and the PolyBayes score. To minimize the number of false positives we included in the assay only SNPs with a PolyBayes score above 99%, with either a minor allele appearing at least twice within four to ten ESTs, or a MAF above 20% when more than ten ESTs were available. Indeed, it is highly unlikely that sequencing errors of two independently sequenced ESTs occur at the same base location. We also excluded SNPs that were surrounded by other polymorphisms in the immediate 60 bases to avoid technical problems due to neighboring polymorphisms. In both cases, chromatograms were visually checked to ensure the quality of the flanking sequences, and we used BLASTN analysis [35] to ensure that *in vitro* and *in silico* SNPs belonged to different genes.

### SNP genotyping array

**Figure 1:**
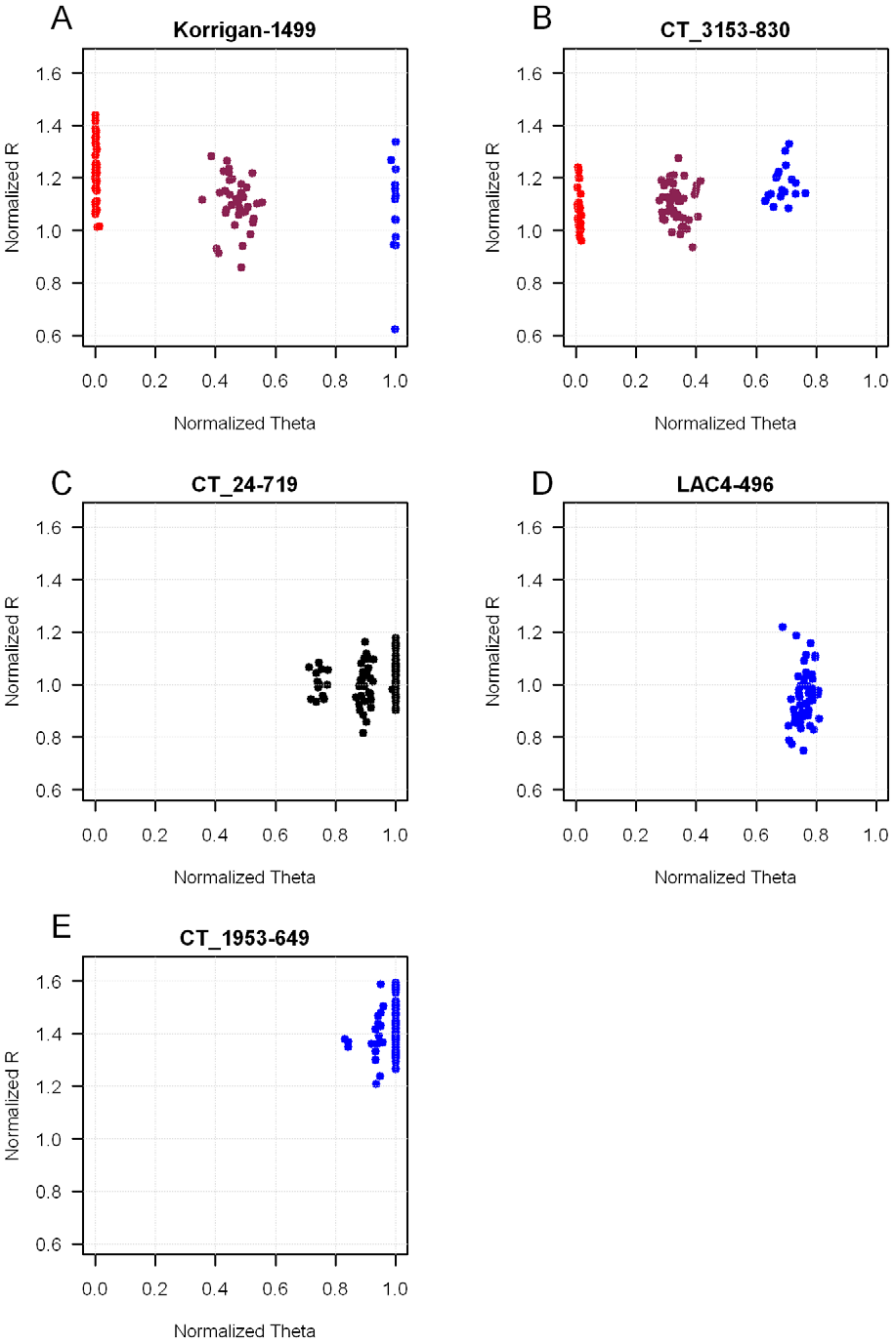
Examples of clustering observed for the *P. pinaster* SNP array. Each dot represents the mean intensity derived from a population of beads for a single sample. The normalized *R* (*y* axis) is the normalized sum of intensities of the two dyes (*Cy3* and *Cy5*), and the normalized *Theta* (*x* axis) is ((2/Л)Tan^−1^ (*Cy5*/*Cy3*)), where a normalized *Theta* value nearest 0 is a homozygous for allele A and a *Theta* value nearest 1 is homozygous for allele B. **A**/ classical pattern with three clusters for a SNP considered as successful and polymorphic. **B** and **C**/ “cluster compression” when both homozygous clusters are closer to each other than expected. In panel **B**, the clustering algorithm is able to distinguish the three clusters and gives a GenTrain score of 0.58, however this kind of pattern was considered as a genotyping failure in our analysis because one of the homozygous cluster normalized *Theta* value does not fall in the [0, 0.1] or [0.9, 1] ranges. In panel **C** the clustering algorithm was not able to distinguish the three clusters because of low separation scores, and the SNP was automatically considered as a genotyping failure because of its low GenTrain score. **D** and **E**/ SNPs interpreted as genotyping failures either because of abnormal *Theta* values (**D**) or because of the presence of subgroups in a cluster (**E**).

The Illumina GoldenGate technology (Illumina Inc., San Diego, CA, USA) was used to carry out the genotyping reactions in accordance with the manufacturer's protocol [36]. To assess the reproducibility of the genotyping assay, 19 DNA samples were duplicated across the different plates. Negative controls were also added to each 96-well plate. Highly multiplexed extension reactions were conducted using 250ng of template DNA per sample. The clustering was realized with the BeadStudio software (Illumina Inc.), and a quality score for each genotype was generated. A GenCall score cutoff of 0.25 was used to determine valid genotypes at each SNP and the SNPs retained had to get a minimum GenTrain score of 0.25, which represents a stringent criterion that is used in human genetic studies [27]. GenCall and GenTrain scores measure the reliability of SNP detection based on the distribution of genotypic classes (AA, AB and BB). Clusters were visually inspected to ensure high quality data (Figure 1). When we observed cluster compression (*i.e.* when the homozygous clusters normalized theta values were not in the [0, 0.1] or [0.9, 1] ranges, as illustrated in Figure 1 B, C and D), we considered that the genotyping failed, as this is likely due to genome redundancy [29]. Indeed, the compression of the BB homozygous cluster towards the AA cluster could result from a paralog gene matching the A allele, increasing the signal for the A dye for both BB and AB genotypes. We also considered as genotyping failures monomorphic SNPs for which clusters could be divided in two or more subgroups such as illustrated in Figure 1E.

### Measuring error rate using pedigree data

We used the breeding population pedigree information (relationships between first and second generation) to detect possible Mendelian Inconsistencies (MIs) between parents and offspring using the PedCheck software [37]. Then, we used the method described in Saunders *et al.* [38] to estimate the genotyping error rate Π from MIs. Genotyping errors (GEs) are not all detectable as MIs, but there is a linear relationship between the GE and the MI counts has shown by Hao *et al.* [39]. The expected number of MIs at a marker (Π.*P_MI_*) in a family in which one or both parents and *m* children have been genotyped can be derived from the marker allele frequency *p* in the studied population, *m* and Π as follows [38]. If only one parent has been genotyped:
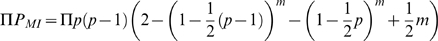
and if both parents have been genotyped
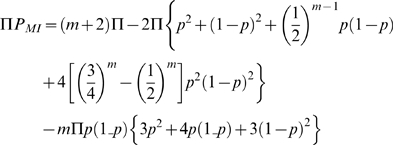
These relationships can be easily generalized to large non-inbred pedigrees and many SNPs, by summation of Π.*P_MI_* over all families and averaging Π over all SNPs. This procedure allows to estimate a *per* SNP as well as a global genotyping error rate [38]. We performed this analysis on 17 unrelated families from the breeding population, using for each marker the allele frequency (*p*) estimated on the Aquitaine G0 genotyping dataset (212 samples).

## Results

### SNP detection and construction of the SNP array

A total of 448 *in vitro* SNPs were detected in the dataset of re-sequenced fragments. Overall 155, 81 and 28 SNPs were discarded because of low functionality scores, neighboring polymorphisms, or because they corresponded to rare variants, respectively. The 184 remaining SNPs included in the assay represented 40 different gene fragments (Table S3).

**Figure 2:**
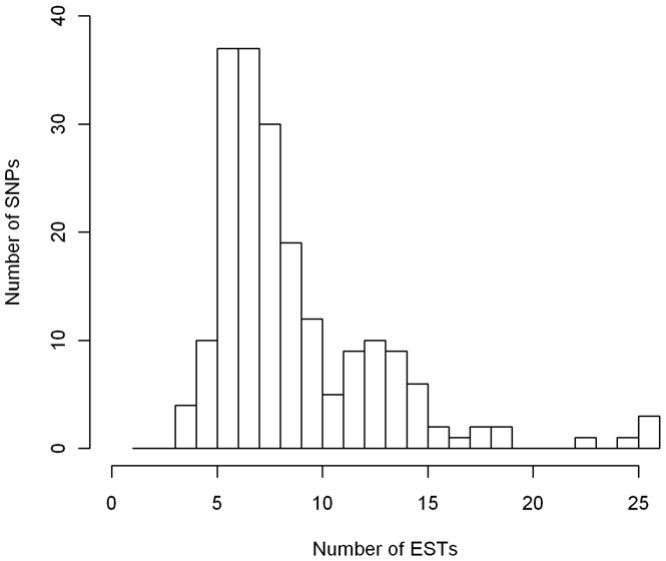
Distribution of the 200 *in silico* SNPs according to the number of ESTs considered for the detection.

Similarly, 9,364 *in silico* SNPs were detected in the unigene set, and we selected 200 of them satisfying our very stringent criteria, *i.e.* PolyBayes and functionality scores, polymorphism proximity, minimum number of ESTs for the detection, MAF and visual validation of the chromatograms. They represented 146 different unigene elements. Figure 2 shows the number of ESTs considered for the detection of the 200 *in silico* SNPs.

### Reproducibility and overall success rate of the SNP assay

No discordance was detected between the 19 replicated samples, *i.e.* the same genotype was observed over the replicates, yielding a reproducibility rate of 100%. For nine polymorphic SNPs we observed cluster compression (as in Figure 1B), and for nine monomorphic SNPs we found either unexpected normalized theta values, or subgroups in a homozygous cluster (as in Figure 1D and 1E, respectively). In those cases we considered that the genotyping failed despite acceptable GenTrain scores.

To measure the global success of the genotyping assay we first estimated the success-rate, which corresponds to the number of SNPs that are successfully genotyped (considering both monomorphic and polymorphic SNPs) divided by the total number of SNPs in the assay, and second the conversion rate, which is the number of polymorphic SNPs divided by the total number of SNPs in the assay, as defined in Fan *et al.* [27]. Among the 384 SNPs analyzed, 257 were successfully genotyped (Table 2), leading to a global success-rate of 66.9%. The minimum GenTrain score observed for these SNPs was 0.53. A total of 60 SNPs were found to be monomorphic in the tested samples, yielding a conversion rate of 51% (Table 2).

**Table 2:** Success rate of the genotyping assay.

Category	Nb of SNPs (*in vitro*/*in silico*)	% of SNPs (*in vitro*/*in silico*)
Failed^1^	127 (50/77)	33% (27%/38.5%)
Monomorphic^2^	60 (22/38)	16% (12%/19%)
Polymorphic^3^	197 (112/85)	51% (61%/42.5%)
Total	384 (184/200)	100% (100%/100%)

1 Failed genotyping, *i.e.* GenTrain score <0.25 or cluster compression.

2 Genotyping successful but monomorphic SNPs.

3 Genotyping successful and polymorphic SNP.

The mean call rate, which is 1 minus the rate of missing data, exceeded 98% at the SNP level and ranged from 73.4% to 93.5% at the sample level for four of the five plates analyzed. It dropped to 77.5% at the SNP level and ranged from 13.8% to 87.5% at the sample level for the fifth plate where we noticed evaporation problems during the genotyping reactions. We found significant differences depending on the origin of the markers: *in vitro* SNPs generally showed significantly higher genotyping-success and conversion rates compared to *in silico* SNPs (+11.5% and +18.5% with χ^2^-test *P*-values of 0.025 and 4.73.10^−4^, respectively).

**Figure 3:**
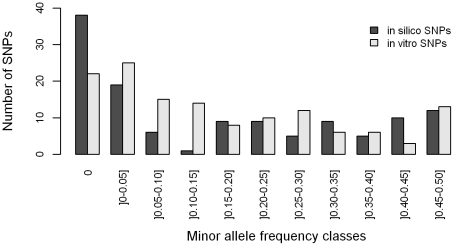
Allele frequency spectrum for 257 successfully genotyped *in vitro* and *in silico* SNPs.

The distribution of allelic frequencies for *in vitro*- and *in silico* SNPs is shown in Figure 3. Among successfully genotyped SNPs, monomorphic loci were twice more abundant for *in silico* SNPs compared to *in vitro* SNPs (30.9% versus 16.4%, respectively). Most of the 22 monomorphic *in vitro* SNPs corresponded to either SNPs that were monomorphic in the Aquitaine sequences (10 SNPs), rare variants (3 SNPs with a MAF below 5% in the Aquitaine sequencing dataset), or were detected on alignments that did not include any sequences from south-western France (3 SNPs). Among the polymorphic SNPs, 35.7% of *in vitro* and 29.4% of *in silico* SNPs corresponded to rare variants (MAF ≤10%) (Figure 3).

### SNP success rate according to SNP functionality score

**Figure 4:**
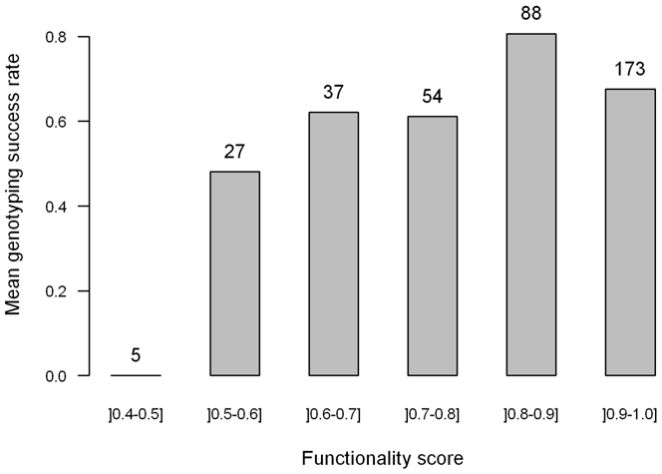
Genotyping success rate according to functionality score for the 384 SNPs of the assay. The number of SNPs in each functionality score class is indicated above each bar.

Prior to the construction of the SNP bead array, a functionality score was calculated for each candidate SNP using the Illumina Assay Design Tool. The higher the score, the more likely will the SNP be successfully genotyped. We could not genotype any of the five SNPs with functionality scores below 0.5, and only 13 of the 27 SNPs with functionality scores between 0.5 and 0.6 (Figure 4). SNPs with a predicted functionality score above 0.6 had a much higher success rate than those below 0.6 (χ^2^-test *P*-value of 0.0019), as found in Pavy *et al.* [23] for white and black spruce. This also agrees with Illumina's recommendations of using only SNPs with a functionality score above 0.6 to ensure a high success rate for the assay.

### Comparison of allele frequency estimated by sequencing and genotyping

Among the 112 polymorphic *in vitro* SNPs of the genotyping assay, 101 were previously identified in alignments containing 10 sequences or more from the Aquitaine population and were used to assess the reliability of allele frequency estimates based on sequencing data. The correlation between marker allele frequencies determined by sequencing and genotyping was ∼0.83 (considering only the 212 unrelated samples from the Aquitaine G0 breeding population) (Figure S1), showing that allelic frequencies estimated by genotyping were generally in the range of those estimated by sequencing.

### Measuring genotyping error rate with pedigree data

For 84 and 81 offsprings of the G1 population, either one or both parental G0 trees were genotyped in the assay. This dataset consisted in 36,991 genotyping datapoints corresponding to 222 samples (165 G1 and 57 G0 trees) genotyped for 188 polymorphic SNPs, after excluding 4,745 missing data. We found a total of 181 Mendelian Inconsistencies (MIs). Most of these errors (75%) appeared in only nine parents-offspring pairs for which the MI rate ranged from 4% to 17%, suggesting laboratory errors (either traceability errors during the controlled pollination, plant material sampling and handling, wet lab experiment, or DNA contamination) rather than genotyping errors. In six cases we assumed that the MIs originated from the offspring genotypes as the parents were involved in other crosses where no MI was found. For the other cases we could not tell parents and offspring MIs apart. Setting aside these possible human errors, 46 MIs were detected for 35,521 genotyping datapoints. MIs were not significantly more abundant for samples presenting low call rates (χ^2^-test *P*-value of 0.51, see also Table S4). To estimate the genotyping error rate Π, we used a subset of 17 unrelated families corresponding to 75 G1 trees and their 26 G0 parents genotyped for 188 polymorphic SNPs (18,261 genotyping datapoints after removing 727 missing data). The observed MI count for this subset was 28, yielding a global mean genotyping error rate Π of 0.54%. At the SNP level, a total of 181 SNPs showed no MIs and thus a null *per* SNP genotyping error rate. Among the seven remaining markers, three showed error rates ranging from 2.9% to 3.3%, and four (two *in vitro* SNPs and two *in silico* SNPs) showed particularly elevated error rates (Π between 16% and 70%). In these cases (distribution of error rates skewed owing to four SNPs with very high error rates), the estimate of the mean error rate tends to be biased upwards [38]. When removing these four SNPs, the observed MI count dropped to 3, leading to a mean error rate Π of 0.06%.

## Discussion

### Data summary

A 384-SNPs GoldenGate genotyping array for *Pinus pinaster* was built from i/ 448 SNPs originally detected in a set of 41 re-sequenced candidate genes (*in vitro* SNPs) and ii/ 9,364 SNPs screened from ESTs (*in silico* SNPs). Two different SNP selection strategies were followed, “depth *vs.* breath of SNP coverage”. For *in vitro* SNPs we aimed at validating as many polymorphisms as technically possible for each fragment (depth), whereas for *in silico* SNPs we aimed at validating few SNPs per unigene in a large number of unigenes (breath). A total of 184 *in vitro* SNPs were chosen on the basis of functionality scores, presence of neighboring polymorphisms, MAF and linkage disequilibrium. Moreover, 200 *in silico* SNPs were selected based on three parameters that proved critical for high validation rate of EST-derived SNPs [18]: the number of ESTs used for SNP detection, the SNP MAF and the quality of SNP flanking sequences. The global success rate of the assay was 66.9% (considering monomorphic and polymorphic SNPs), and a conversion rate of 51% was achieved (considering only polymorphic SNPs). *In vitro* SNPs showed significantly higher genotyping success (+11.5%, *P*-value 0.025) and conversion (+18.5%, *P*-value 4.73.10^−4^) rates than *in silico* SNPs. The functionality score estimated for each SNP, which in our case could not account for sequence redundancy in the genome, showed a significant relationship with success of genotyping. The reproducibility of the assay was very good (100%, based on 19 replicated genotypes), and the genotyping error rate very low (0.54%, dropping down to 0.06% when removing four SNPs showing elevated error rates).

### Conversion rates of *in vitro* and *in silico* SNPs for *Pinus pinaster*

Data obtained from the GoldenGate assay reported in this paper suggest that the bead array technology is suitable for the complex and large genome of *P. pinaster*: 66.9% of the SNPs were translated into easily interpreted genotypic clusters. This success rate is similar to that observed for *Pinus taeda* [66.9%, 24], but lower than that observed for *Picea glauca* or *Picea mariana* [78.5% and 81.1% respectively when considering polymorphic and monomorphic SNPs, 23]. So far, two main causes have been invoked in the literature for explaining genotyping failures in GoldenGate assays for non-model species. First, the partial knowledge of large and redundant genomes can be a limiting factor to design an efficient SNP genotyping assay. Indeed, flanking sequences cannot be fully validated for locus specificity and the possible presence of repetitive elements [23], [27], [34]. Secondly, the sample size used for SNP discovery in species presenting a high level of nucleotide diversity may be too small, possibly leading to the presence of undetected SNPs within priming sites when larger sample of trees are genotyped [24]. In the case of *Pinus pinaster*, both hypotheses can be examined: we reached a 79.6% success rate when considering a group of 103 *in vitro* SNPs that were detected on more than 30 individuals from the Aquitaine population, which is similar to that observed in *Picea* species [78.5% and 81.1% in *P. glauca* and *P. mariana*, respectively, 23]. The rate dropped to 55.8% for another group of 43 *in vitro* SNPs detected on 10 to 30 samples. We checked that this difference in success rates was not due to differences between allelic frequency distributions in both groups (data not shown). This significant difference (χ^2^-test *P*-value of 0.006) suggests that the sample size of the SNP discovery panel has a large impact on the conversion rate. However, the high conversion rate achieved using SNPs from well characterized DNA regions (79.6%) still does not reach that reported for human [>91% in 27,40,41,42]. As discussed in Pavy *et al.* [23], the megagenome of conifers may hinder the development of specific probes for the assay. The nine cases of cluster compression detected in our assay support this hypothesis. The shift of a homozygous cluster toward the other one has previously been observed for a SNP in a gene presenting a nearly identical paralog in soybean, and is likely the sign of the targeted-sequence redundancy [29].

We found a significant difference between *in vitro* SNP and *in silico* SNP conversion rates, a lower rate being observed for *in silico* SNPs. According to Wang *et al.* [18], genotyping failures in ESTs-derived SNPs may come either from sequencing errors that lead to the identification of false-positive SNPs (pseudo-SNPs), from low quality of SNPs flanking sequences, or from the presence of an exon-intron junction near the SNP of interest. In our study, the selection of false-positive SNPs should have been prevented by the use of trace data for SNP detection [33], and a set of stringent criteria including MAF and contig size. Indeed, Wang *et al.* [18] achieved a 70.9% conversion rate for catfish *in silico* SNPs detected on at least four sequences and with a minor allele present twice, against a rate of 33.3% for SNPs detected on four or fewer sequences with minor allele present only once. In our case, chromatograms have also been checked to ensure high-quality of flanking sequences for primer design, but the presence of undetected polymorphisms in these regions is likely as most SNPs were detected on only ten ESTs or less (Figure 2). We could not confirm whether or not *in silico* SNPs were located at exon-intron borders, as we lack a fully sequenced conifer genome to compare with. The presence of introns has been identified as a major cause for *in silico* SNP genotyping failures [18], and may explain the conversion rate difference between *in vitro* (revealed from genomic DNA sequences) and *in silico* (discovered from mRNA sequences) SNPs. We previously defined the conversion rate as the number of polymorphic SNPs divided by the total number of SNPs in the assay. Since monomorphic loci were twice more abundant for *in silico* SNPs than for *in vitro* SNPs, this also partly explains their lower conversion rate. Indeed, the EST database used for *in silico* SNP detection included sequences from samples of various origins (Corsica, Spain and Aquitaine, see Table 1), leading probably to the detection of a small quantity of population-specific *in silico* SNPs. On the other hand, more than 80% of *in vitro* SNPs originated from individuals collected in the Aquitaine provenance region (Table S3), *i.e.* the same material than the genotyped population. Therefore one should remain careful to check and control that the discovery panel for SNPs, whether *in silico* or *in vitro*, matches as closely as possible the genotyped plant material in order to improve the conversion rate. When material of different origins needs to be genotyped in a species showing significant population structure, the genotyping array can only be a compromise, and this situation is likely to be common with the development of arrays including thousands of SNPs. Identifying and better accounting for the provenance of sequences in EST databases when choosing *in silico* SNPs thus seem crucial and is more and more documented either in unigene assemblies or in SNP databases (see for example the NCBI database available at http://www.ncbi.nlm.nih.gov/SNP/). This information not being available upfront in the unigene that we used for *in silico* SNPs discovery, we had overlooked its influence initially, but have been integrating it in future studies.

Surprisingly, six *in vitro* SNPs were found monomorphic on the genotyped trees, while they were detected as polymorphic loci with intermediate frequency estimates in the re-sequenced haploid panel from the Aquitaine population. Given that we are confident that they were not sequencing artifacts, this observation could be explained by either the lack of amplification of one allele due to polymorphism in the priming site, the presence of gametophyte selection against deleterious mutations (as sequences were obtained from haploid megagametophytes while genotyping was performed on diploid DNA), or the general complexity of the *pine* genome as previously discussed. In the latter case, the distinction between genotyping reaction failures and monomorphic SNPs is not obvious. In this study we decided to discard nine monomorphic SNPs with acceptable GenTrain scores but showing either subgroups in the homozygous cluster, or normalized theta values departing from the classical 0/1 values for an homozygous locus. These patterns might be particular forms of cluster compression (shift of the BB cluster toward the AA cluster as illustrated in Figure 1D, or putative shift of the AA and AB clusters toward the BB cluster, Figure 1E). The main quality metrics for SNP assays (GenCall and GenTrain scores) measure the capacity to group samples into genotypic clusters, but to our knowledge no study have established yet the ability of genotype calling algorithms to tell apart failed reactions from monomorphic markers, or to detect cluster compression. Even if geneticists are generally not interested in failed or monomorphic markers, as they do not carry any information, detecting cluster compression would be very useful for non-model species. Markers presenting such patterns should not be used in highly heterozygous populations such as mapping pedigrees, as the heterozygous cluster is often indistinguishable from one or both homozygous ones [29].

### Genotyping error rate

All large genotype datasets have errors that can be either due to sample mishandling, failures of analysis algorithms, or simply biochemical anomalies. Inclusion of incorrect data in genetic analysis can lead to an inflation in genetic map distances [43], an increase in type I error and/or a decrease in statistical power in association studies [44], [45], or to biased estimates of linkage disequilibrium [46] and other allele-frequency related parameters [47]. Errors in a dataset can be detected either by comparing genotypic information obtained from different technologies or by using Mendelian Inconsistencies (MIs) in family-based samples. In this study, we identified nine samples that concentrated 75% of all the observed MIs, which was interpreted as human errors. Sample mishandling has already been identified as a main issue during the genotyping process [47], [48], and could be reduced by the use of traceability systems such as Laboratory Information Management Systems (LIMs), quality insurance standards, and reduced human manipulation, according to the automation possibilities.

Using pedigree information of unrelated families, we also estimated a *per* SNP genotyping error-rate [38], which provides complementary information and helps to identify error-prone loci that can be removed from the study to increase its reliability. For example, the mean error rate per locus dropped from 0.54% to 0.06% when removing the four (out of 188) polymorphic loci that had the highest error rate. These genotyping error-rates are in the range of those recently reported for tetraploid and hexaploid wheat [0% and 1%, respectively, 30]. Unfortunately, genotyping error-rates have seldom been reported for GoldenGate assays in non-model species. While this technique already proved accurate for human, the species for which it was developed [27], its reliability in the complex genomes of plants should be estimated before extensive use. If moderate error rates can be tolerated in cases such as QTL studies involving frequent alleles [44], or identical by descent-based analyses when considering a large number of markers [38], conversely low error rates can be dramatic in association-mapping studies [49]. Once the genotyping error-rate has been estimated, statistical tools that account for it have been developed for linkage analysis [50], family or population-based association mapping [51], [52], [53].

### Conclusion and perspectives

In this study, we demonstrated that ESTs provide a resource for SNP identification in non-model species, which do not require any additional bench work and little bioinformatics analysis. However, the time and cost benefits of *in silico* SNPs are counterbalanced by a lower conversion rate than *in vitro* SNPs. This drawback is acceptable for population-based experiments (in our study, a 42.5% conversion rate was achieved for *in silico* SNPs, compared to 61% for *in vitro* SNPs), but could be dramatic in experiments involving samples from narrow genetic backgrounds. For example, Eckert *et al.* [24] only reached an 18.2% conversion rate in a *P. taeda* mapping pedigree, using *in vitro* SNPs from a database that did not include any sequences of the parental lines of the mapping population. In addition, we showed that both the visual inspection of genotyping clusters and the estimation of a *per* SNP error rate should help identify markers that are not suitable to the GoldenGate technology in species characterized by a large and complex genome.

Recently, a larger-scale SNP-array was designed for maritime pine, comprising 1,536 SNPs (826 *in vitro* SNPs, including 560 SNPs detected from re-sequenced amplicons provided by David Neale, UC Davis, CA, USA, http://dendrome.ucdavis.edu/crsp/, and 710 *in silico* SNPs selected with the same criteria as in this study). This second generation SNP-array will be used to establish a species consensus map based on the analysis of seven pedigrees, and for association mapping for a series of traits (biomass production, wood and end-use properties, drought stress resistance) measured on clonal and progeny tests on the first and second breeding populations.

## Supporting Information

Figure S1 Correlation between allele frequencies estimated by sequencing and genotyping for 101 *in vitro* SNPs. The plain lines and dashed lines correspond to the 95% bootstrap confidence intervals for allele frequencies estimated on 20 or 50 samples, respectively.
(6.54 MB TIF)

Table S1 NCBI ss accession numbers for *in vitro* and *in silico* SNPs that were polymorphic in the assay.
(0.03 MB XLS)

Table S2 List of the 41 candidate genes used for *in vitro* SNPs detection and associated projects.
(0.03 MB XLS)

Table S3 List of the 184 *in vitro* SNPs and their frequencies in the total sequencing dataset, in the Aquitaine sequencing dataset and in the genotyped samples.
(0.06 MB XLS)

Table S4 Call rate classes of the genotyped samples.
(0.02 MB XLS)
