# Supplementary figures and images for: In Vitro vs In Silico Detected SNPs for the Development of a Genotyping Array: What Can We Learn from a Non-Model Species?
Source: PLoS One. 2010 Jun 9;5(6):e11034. doi: 10.1371/journal.pone.0011034 (PMC2882948; doi:10.1371/journal.pone.0011034)

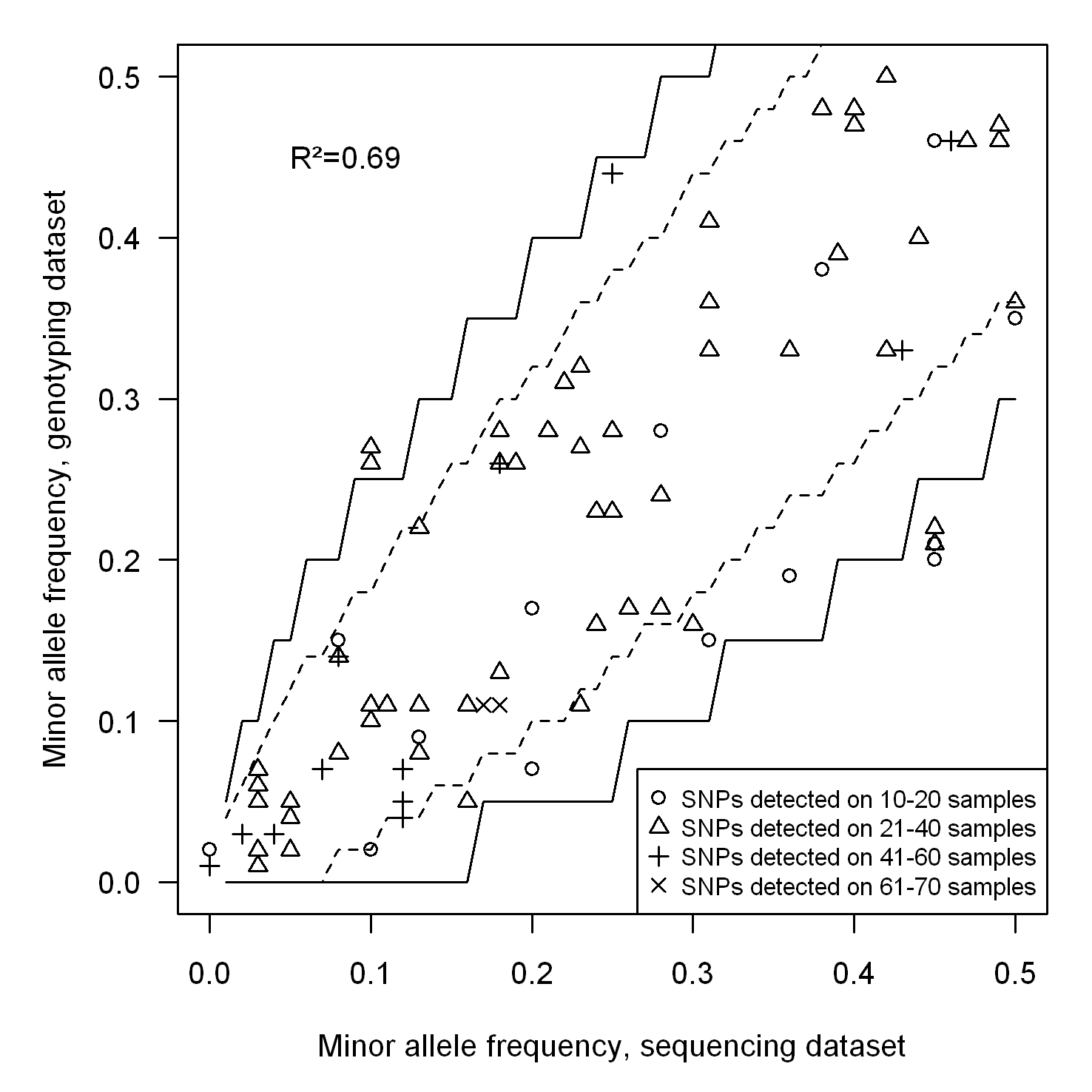

Supplement: Figure S1 — Correlation between allele frequencies estimated by sequencing and genotyping for 101 in vitro SNPs. The plain lines and dashed lines correspond to the 95% bootstrap confidence intervals for allele frequencies estimated on 20 or 50 samples, respectively. (6.54 MB TIF) [file pone.0011034.s001.tif]
